# Early Keratometric and Scheimpflug Topographic Stabilization after Pterygium Excision with Conjunctival Autograft: A Prospective Cohort Study

**DOI:** 10.22336/rjo.2026.11

**Published:** 2026

**Authors:** Farhat Siddiqui, Raj Nath Singh Kushwaha, Perwez Khan, Ankita Srivastava

**Affiliations:** 1 Ophthalmology Department, Era’s Lucknow Medical College and Hospital, Lucknow, Uttar Pradesh, India; 2 Jalaun Government Medical College, Uttar Pradesh, India; 3 Ophthalmology Department of GSVM Medical College, Kanpur, Uttar Pradesh, India; 4 Ophthalmology Department, Government Medical College, Amethi, Uttar Pradesh, India

**Keywords:** pterygium, conjunctival autograft, keratometry, Scheimpflug imaging, corneal astigmatism, BCVA = Best-Corrected Visual Acuity, D = Diopter, RJO = Romanian Journal of Ophthalmology, UV = Ultraviolet

## Abstract

**Objective:**

To evaluate early corneal stabilization following pterygium excision with conjunctival autograft, using keratometry and Scheimpflug imaging, and to compare outcomes with recent evidence.

**Methods:**

In this prospective cohort of 70 eyes (February 2019-February 2020), primary nasal pterygium excision with conjunctival autograft was performed using either fibrin glue (n = 35) or Autologous plasma (n = 35). Keratometric and Scheimpflug parameters [corneal astigmatism, index of surface variance (ISV), index of vertical asymmetry (IVA), maximum keratometry (Kmax), posterior corneal astigmatism] were assessed preoperatively, and at 1- and 2-month follow-up. Outcomes were compared using paired t-tests and subgroup analysis.

**Results:**

Mean keratometric astigmatism decreased from 1.35 ± 1.12 D preoperatively to 0.71 ± 0.65 D at 2 months (p < 0.001). Scheimpflug-derived astigmatism reduced from 1.22 ± 0.95 D to 0.89 ± 0.71 D (p < 0.001). ISV improved from 42.6 ± 6.4 to 33.2 ± 4.9 (p < 0.01), IVA from 0.38 ± 0.12 to 0.29 ± 0.10 (p < 0.01), and Kmax decreased modestly (p < 0.05). Outcomes were comparable between glue and suture groups (p > 0.05). Recurrence occurred in 3 eyes (4.3%).

**Discussion:**

The present study demonstrated early stabilization of keratometric and Scheimpflug parameters following pterygium excision with conjunctival autograft, suggesting that significant corneal remodeling occurs within the early postoperative period.

**Conclusions:**

Pterygium excision with conjunctival autograft yields a significant early reduction in astigmatism and an improvement in corneal surface regularity by 2 months, independent of fixation technique. These results support early refractive rehabilitation and spectacle planning after surgery.

## Introduction

Pterygium is a fibrovascular conjunctival proliferation encroaching onto the cornea, strongly associated with ultraviolet (UV) exposure, dust, and wind. It is prevalent in tropical and subtropical regions, particularly within the “pterygium belt” [1,2] and disproportionately affects outdoor workers. In addition to cosmetic concerns, pterygium induces visually significant with-the-rule astigmatism [3,4] through localized corneal flattening, the degree of which correlates with lesion size and vascularity.

Surgical excision remains the treatment of choice. The conjunctival autograft technique, first described by Kenyon et al. [5], has become the gold standard, offering lower recurrence rates [6] and more favorable cosmetic outcomes than bare scleral excision. Graft fixation methods vary (sutures, fibrin glue [7,8], autologous blood), yet clinical outcomes are generally comparable.

Objective assessment of corneal curvature is crucial in quantifying the visual impact of pterygium and the benefits of surgery. Manual keratometry provides quick central curvature readings, but advanced modalities like Scheimpflug [9] imaging (Pentacam) allow comprehensive anterior and posterior corneal analysis, including indices of regularity (ISV, IVA, CKI) and keratometric power (Kmax).

Several studies have examined keratometric outcomes [10] after pterygium surgery, but few have combined keratometry and Scheimpflug [9] imaging in the same cohort with prospective follow-up. Moreover, most reports focus on long-term recurrence [6], whereas early refractive stabilization — critical for spectacle prescription and planning for cataract surgery — remains underexplored.

The present study prospectively evaluates early keratometric and topographic stabilization after pterygium excision with conjunctival autograft, comparing fixation techniques and aligning findings with recent global evidence.

## Materials and methods

### Study Design and Ethical Approval

This was a prospective, interventional, hospital-based study conducted in the Department of Ophthalmology at GSVM Medical College, Kanpur, from February 2019 to February 2020. Institutional Ethics Committee approval (EC/BMHR/2020/43) was obtained, and all participants provided written informed consent. The study adhered to the Declaration of Helsinki.

### Sample Size

Based on an expected mean astigmatism reduction of 0.5 D (SD 0.9), 63 eyes were required (α = 0.05, 80% power). To account for dropouts, 70 eyes were included.

### Eligibility Criteria

Inclusion: Adults 20-50 years, primary nasal pterygium ≥ 2 mm onto cornea, clear cornea, no prior ocular surgery. Exclusion: Recurrent or pseudo-pterygium, symblepharon, corneal opacity, coexisting ocular pathology, systemic disease affecting healing.

Pterygium Grading: Grade II: > 2 mm – ≤ 4 mm; Grade III: > 4 mm, not involving visual axis.

### Preoperative Evaluation

BCVA (Snellen → logMAR), slit-lamp exam, auto-refraction, manual keratometry, Scheimpflug imaging (Pentacam HR: anterior/posterior astigmatism, ISV, IVA, Kmax), fundus exam, systemic screening.

### Surgical Technique

All surgeries were performed by a single surgeon under peribulbar anesthesia. Standard excision of the pterygium head and fibrovascular tissue was followed by harvesting a superior bulbar conjunctival-limbal autograft. The graft was secured either with fibrin glue [7,8] (n = 35) or autologous plasma (n = 35).

### Postoperative Regimen

Topical moxifloxacin and dexamethasone (tapered) with lubricants for 4 weeks.

### Follow-up

Day 1, Week 1, Month 1, Month 2. Keratometry and Scheimpflug [9] were repeated at 1 and 2 months.

### Outcome Measures

Primary: Change in corneal astigmatism (D). Secondary: ISV, IVA, Kmax, posterior astigmatism, complications, recurrence [6].

### Statistical Analysis

SPSS v20.0 was used. Continuous data as mean ± SD. Paired t-test for pre- vs. postoperative comparisons; subgroup analysis (glue vs. suture). p < 0.05 was considered significant.

## Results

### Demographics

Seventy eyes of 70 patients were analyzed. Mean age was 37.2 ± 1.7 years (range: 25-50), with 42 males (60%) and 28 females (40%). The right eye was affected in 38 (54.3%) and the left in 32 (45.7%). Most patients (n = 56; 80%) reported significant occupational UV exposure, predominantly among farmers and outdoor laborers. All demographic and baseline characteristics are shown in **Table 1**.

**Table 1:** Demographic and baseline characteristics (n=70)

Parameters	Value
Age (years)	37.2 ± 1.7
Gender	Male 42 (60%), Female 28 (40%)
Laterality	Right 38 (54.3%), Left 32 (45.7%)
Occupational UV exposure	56 (80%)
Grade	II: 43 (61.4%), III: 27 (38.6%)

### Pterygium grading

Grade II in 43 eyes (61.4%) and Grade III in 27 eyes (38.6%).

### Keratometric Astigmatism

Mean keratometric astigmatism decreased significantly from 1.35 ± 1.12 D preoperatively to 0.71 ± 0.65 D at 2 months (p < 0.001).

### Scheimpflug Topographic Parameters

Scheimpflug-derived astigmatism reduced from 1.22 ± 0.95 D to 0.89 ± 0.71 D at 2 months (p < 0.001). Indices of corneal regularity also improved: ISV: 42.6 ± 6.4 → 33.2 ± 4.9 (p < 0.01); IVA: 0.38 ± 0.12 → 0.29 ± 0.10 (p < 0.01); Kmax: 44.7 ± 2.2 → 43.8 ± 1.9 D (p < 0.05). Posterior corneal astigmatism showed no significant change (p = 0.08).

Mean keratometric and Scheimpflug-derived astigmatism showed a significant reduction following pterygium excision, as summarized in **Table 2**.

**Table 2:** Corneal astigmatism before and after pterygium excision (keratometry and Scheimpflug imaging)

Measurement method	Pre-operative (Mean ± SD)	Post-operative 2 months (Mean ± SD)	p-value
Keratometry	1.35 ± 1.12 D	0.71 ± 0.65 D	<0.001
Scheimpflug imaging	1.22 ± 0.95 D	0.89 ± 0.71 D	0.0001

### Subgroup Analysis (Glue vs. Suture)

Both fixation techniques demonstrated comparable reductions in corneal astigmatism, with no statistically significant difference between the glue and suture groups (p > 0.05). The mean reduction in astigmatism was 0.62 ± 0.35 D in the glue group and 0.58 ± 0.41 D in the suture group. Similarly, indices of corneal surface regularity showed no significant intergroup differences, with ISV reduction of 8.2 ± 3.4 in the glue group and 7.9 ± 3.7 in the suture group (p = 0.72). No significant difference was observed in IVA or Kmax values between the two groups. The recurrence rate was also comparable, with 4.1% in the glue group and 4.5% in the suture group (p = 0.88). These findings indicate that both fixation techniques provide similar clinical and refractive outcomes **(Table 3)**.

**Table 3:** Comparison between glue and suture fixation techniques

Parameter	Glue (n = 24) Mean ± SD	Suture (n = 22) Mean ± SD	p-value
Astigmatism reduction (D)	0.62 ± 0.35	0.58 ± 0.41	0.64
ISV reduction	8.2 ± 3.4	7.9 ± 3.7	0.72
Recurrence (%)	1 (4.1%)	1 (4.5%)	0.88

### Complications and Recurrence

Recurrence: 3 eyes (4.3%) by 2 months; Graft edema: 2 eyes (2.8%), resolved with topical steroids. No cases of infection, graft loss, or persistent epithelial defect were observed, as summarized in **Table 4**.

**Table 4:** Post-operative complications

Complication	Number (%)
Recurrence	3 (4.3%)
Graft edema	2 (2.8%)
Infection	0 (0%)
Persistent epithelial defect	0 (0%)

## Discussion

### Early Keratometric and Topographic Stabilization

Our study demonstrates a significant early reduction in corneal astigmatism following pterygium excision with conjunctival autograft. Mean keratometric astigmatism decreased from 1.35 D to 0.71 D, while Scheimpflug-derived astigmatism reduced from 1.22 D to 0.89 D by 2 months. These findings align with earlier studies that established pterygium as a cause of high with-the-rule astigmatism and showed improvement after excision [10].

### Fixation Method Does Not Alter Outcome

Both fibrin glue and sutures produced comparable outcomes in terms of keratometric reduction and surface regularity. This indicates that the fixation technique may be chosen based on the surgeon’s preference, cost, and availability without compromising refractive stability. These results mirror prior trials and reviews that found no significant differences in outcomes between fixation methods [7].

### Improvements in Corneal Regularity

Scheimpflug imaging demonstrated marked improvement in ISV and IVA, indicating enhanced surface symmetry and optical quality after surgery. While earlier keratometry- and topography-based studies focused solely on astigmatism reduction, our study provided objective evidence of improved regularity indices [10,11].

### Recurrence and Safety

A recurrence rate of 4.3% was noted at 2 months, with no major complications. This is consistent with recurrence rates of 2-10% reported in long-term autograft studies [5,6]. The low early recurrence highlights the safety and effectiveness of the procedure.

### Clinical Implications: Early Refractive Planning

The demonstration of corneal stability within 2 months supports early spectacle correction and cataract biometry, potentially as early as 6-8 weeks after surgery. This contrasts with earlier recommendations to wait ≥ 3 months, which were based solely on keratometry [10]. More recent evidence also supports early refractive planning once stability is demonstrated [11].

### Novelty and Comparison with Other Studies

The novelty of our study lies in the dual-modality assessment of outcomes, combining manual keratometry with Scheimpflug-derived indices (ISV, IVA, Kmax, posterior astigmatism). Earlier studies primarily relied on keratometry or Placido-disc topography, providing fewer comprehensive data on surface regularity.

Compared with older literature, our findings confirmed the classical understanding that larger pterygia induce greater astigmatism and that excision improves corneal curvature [3,4]. This historical evidence remains highly relevant as the foundation of modern surgical indications.

Compared with recent studies, our results align closely with Sen et al. (2023), who reported significant astigmatism reduction across graft fixation methods [1,1], and Oltulu R et al. (2013), who showed anterior and posterior remodeling with Pentacam [1,2]. Our study added to this by specifically analyzing surface regularity indices. Similarly, anterior segment optical coherence tomography–based studies have demonstrated significant corneal topographic alterations in pterygium, supporting the findings of the present study [13] and paralleling our findings.

Thus, our study bridges older foundational evidence with modern imaging-based assessments, strengthening the case for early optical rehabilitation after pterygium surgery.

### Limitations and Future Directions

The main limitations of our study were the short follow-up and the inclusion of two fixation methods, although subgroup analysis found no significant differences. Longer follow-up and randomized designs are needed to confirm long-term recurrence and refractive stability. Future research incorporating swept-source OCT and AI-based topography may provide deeper insights into corneal biomechanics and changes in the posterior surface [1,3].

## Conclusion

Pterygium excision with conjunctival autograft provides early and significant reductions in corneal astigmatism and improves surface regularity, as demonstrated by both keratometry and Scheimpflug imaging. By 2 months, the cornea has achieved measurable stability, enabling earlier refractive correction and spectacle planning. Outcomes are comparable whether the graft is secured with sutures or fibrin glue, offering flexibility in surgical practice. While recurrence rates were low in our short-term series, longer follow-up is necessary to assess long-term stability. These findings support early optical rehabilitation following pterygium surgery and highlight the value of dual-modality corneal assessment in routine clinical care.
